# Biomechanical evaluation of different fixation techniques in the rotation of the maxillary occlusal plane after Le Fort I osteotomy

**DOI:** 10.4317/jced.62324

**Published:** 2024-12-01

**Authors:** Danilo de Moraes Castanha, Arthur Alves Thomaz de Aquino, Thalles Moreira Suassuna, Eduarda Gomes Onofre de Araújo, Fábio Andrey da Costa Araújo, Emanuel Sávio de Souza Andrade

**Affiliations:** 1 Faculty of Dentistry of Pernambuco, University of Pernambuco, 50100130, Recife, Pernambuco, Brasil

## Abstract

**Background:**

To analyze the biomechanical and functional characteristics of different maxillary fixation techniques after Le Fort I osteotomy and occlusal plane rotation, using the finite element method to simulate the mechanical behavior of three different osteosynthesis approaches.

**Material and Methods:**

This is a virtual experimental study carried out using finite element analysis to compare three different osteosynthesis techniques after Le Fort I osteotomy and rotation of the maxillary occlusal plane. Three configurations were tested: four-point fixation with “L” plates (C1), two-point fixation with “L” plates (C2), and two-point fixation with pre-modeled Lindorf plates (C3).

**Results:**

The analysis indicated that set C1 showed maximum displacement in the anterior region, while C2 and C3 showed displacement in the posterior region. The von Mises stresses revealed that C1 exceeded the yield limit of titanium, indicating potential failure. On the other hand, C2 and C3 showed a more balanced distribution of stresses within acceptable limits.

**Conclusions:**

The fixation techniques with fewer points (C2 and C3) proved to be as effective, or even superior, to the traditional four-point fixation method (C1), offering better stress distribution and lower mechanical stress.

** Key words:**Le Fort osteotomy, Internal fixation, Finite elements, Biomechanics.

## Introduction

Le Fort I osteotomy is an established technique for correcting dentofacial deformities involving the middle third of the face. Through surgical movement of the maxillae, it restores the normal occlusal relationship and can be used in aesthetic and functional disorders, such as in the management of patients with obstructive sleep apnea (1). Osteotomy conceptually divides the jaws into different segments, allowing three-dimensional mobilization and repositioning according to surgical planning (1,2).

Once the segments have been mobilized, they need to be fixed in the planned position. Initially, osteosynthesis was carried out using steel wires and a maxillo-mandibular block. Over time, fixation materials and methods have undergone numerous evolutions, with the concept of sTable internal fixation being considered one of the main advances in the field. The use of internal devices (plates and screws) juxtaposed to bone structures enables better jaw positioning during the transoperative period, early return to function and better long-term stability (2,3).

Currently, most surgeons use titanium miniplates for maxillary osteosynthesis. For this purpose, it is conventional to distribute four miniplates across the nasomaxillary and zygomatic-maxillary pillars. However, following the trend of modern surgery, with the aim of simplifying the procedure, making it technically faster and less invasive, some authors have proposed modifications to this method. The main change is to reduce the amount of osteosynthesis material. The proposal is to dispense with the fixation of the posterior segment (zygomatic-maxillary pillar) (2,4,5,6). This is expected to significantly reduce surgical time and post-operative discomfort (6).

With the changes to the fixation of maxillae repositioned using the Le Fort I osteotomy technique, questions have also arisen. The questions are mainly about the stability of movement, the ideal location of the plates, the number and size of plates and screws needed for adequate osteosynthesis and the consequent satisfactory surgical result. A few studies have attempted to provide answers to these questions. Some studies have shown satisfactory results when using two internal fixation points, one on each nasomaxillary pillar. Mavili *et al*. (2009) (6) carried out a study of patients undergoing bimaxillary orthognathic surgery with anterior fixation using L-shaped plates with two screws on each side of the maxillary osteotomy and an intermaxillary elastic block started 48 hours postoperatively and maintained for 2-4 weeks. At the end of the follow-up period, accepTable stability in maxillary positioning was observed.

Some manufacturers have developed pre-modeled and/or customized internal fixation systems in order to allow the surgeon to be more agile during surgery, as well as to avoid material fatigue during the adaptation process in the osteomized segments. In 2020, Susarla *et al*. (2020) (4) analyzed the stability of two-point fixation in maxillary orthognathic surgeries using a pre-modeled fixation system with 4 screws on each side of the osteotomy, observing no dimensional changes during the follow-up period.

However, there are still some gaps in the literature, especially regarding the biomechanical implications of using the different methods proposed for maxillary fixation in orthognathic surgery and the biomechanical capacity of these methods in relation to different maxillary movements. A lower-cost alternative for evaluating the different mechanical stresses of a structure is analysis using the finite element method, which can provide details of the system’s mechanical stresses. It also allows functional parameters to be simulated, making it an analytical tool in craniofacial biomechanical studies. The method is able to provide an assessment of the areas of greatest stress, both in the bone tissue and on the surfaces of the osteosynthesis material. The aim was therefore to study the biomechanical and functional characteristics of different maxillary fixation techniques after Le Fort I osteotomy and occlusal plane rotation using finite element analysis.

## Material and Methods

This is an experimental study carried out in a virtual environment, focusing on the creation of virtual models corresponding to different movements and means of osteosynthesis using finite elements. Once the computer model had been created, the biomechanical capacity of different osteosynthesis techniques in different jaw movements was assessed.

The movement tested was a 3° clockwise rotation of the maxillary occlusal plane centered on the upper first molar. Three different osteosynthesis methods were tested for all movements:

● 4-point fixation using 4 L-shaped plates from the 1.5 system positioned on the nasomaxillary and zygomaticomaxillary pillars with 4 screws (5mm) in each plate, set 01 (C1);

● 2-point fixation using 2 L-shaped plates from the 2.0 system positioned on the nasomaxillary pillars with 4 screws (5mm) in each plate, set 02 (C2);

● 2-point fixation using 2 pre-shaped Lindorf plates positioned on the nasomaxillary pillars with 10 screws (5mm) in each plate, set 03 (C3).

The geometric models were built using a helical computed tomography scan of the face of a patient with maxillary hypoplasia. The DICOM (Digital Imaging and Communications in Medicine) file was exported to virtual surgical planning software (Dolphin v.11.7, Chatsworth, CA, USA) in which the Le Fort I osteotomy was simulated, with the previously established movement groups. After the virtual simulation of the osteotomy and surgical movements, the three-dimensional models were transferred to the graphic design software Meshmixer (Autodesh Ind. - San Rafael, California, USA) in order to adapt the format, and then transferred to the finite element analysis software ANSYS (Ansys Inc., Houston, Texas, USA).

The computer models of the titanium plates and screws were made using the Blender graphic design software (Blender Institute - Buikslotermeerplein, Amsterdam, Netherlands), using the files of the scans of the plates and screws made by the Einstar 3D Scanner (Shining 3D - San Leandro, California, USA), based on physical samples from Tóride (Tóride Ind. e Com. Ltda. - Mogi Mirim, São Paulo, Brazil), as used in previous studies (7,8).

In a virtual environment using CAD tools from the ANSYS software (Ansys Inc., Houston, Texas, USA), the virtual models obtained from the surgical simulations were associated with the virtual models of the osteosynthesis material with the characteristics described above. The models were analyzed according to the characterization of the components, using Young’s modulus of elasticity and Poisson’s ratio. Young’s modulus of elasticity for cortical bone was 18,600 MPa, while for medullary bone it was 1,860 MPa. With regard to Poisson’s ratio, the reference value for medullary and cortical bone was 0.34, in line with characterization studies carried out previously (7-9). For the titanium alloys, the Young’s modulus of elasticity was 103,000 MPa and the Poisson’s ratio was 0.34, according to the property information provided by the manufacturer.

Once the different components of the study models had been characterized, the occlusal load application sites and movement restrictions were defined based on the functional characteristics of the maxilla. After defining the parameters, the surface stresses were analyzed for each model. In order to use imaging and computational analysis of models developed using information technology in this study, approval was obtained from the research ethics committee (CAAE: 72314323.0.0000.5188). The study also complies with the Declaration of Helsinki.

## Results

In this study, 3 finite element models were built, consisting of a single maxilla model, with 3 osteotomy fixation methods using different miniplate models. Thus, 3 geometric models were the objects of study in this study.

-Geometric modeling

-Image from CT archive

The tomographic file in 1 mm thickness of DICOM-type slices, with the preoperative purpose of a patient with maxillary hypoplasia. The software Dolphin was used to simulate the Le Fort I osteotomy - with the previously established movement, thus generating two segments - one proximal (articulated with the skull) and one distal (disarticulated from the skull).

The computer model of the maxilla was built and saved as an STL (Standard Tessellation Language) file, which was imported into the Meshmixer software.

Initially, the model was edited while still in the facet format (originals from an STL file) to eliminate holes and regions with some non-conformities, correcting parts that had not been detected in the tomographic images - anterior wall of the maxillary sinuses, infraorbital foramina, bracket surfaces in the buccal regions of the teeth in both arches, as well as the removal of non-functional regions for computer calculations, with only the middle thirds of the face being present, with the exception of the posterior portions - the maxillary sinuses, ethmoid sinuses and orbital contents.

-Images of plates and screws

Images obtained by scanning titanium plates and screws were used. The opacity of the metal surfaces of the devices had to be treated by adding a thin layer of polyvinyl acetate solution to the surface in order to reduce the reflection intrinsic to the metal surface, as well as using a rotating base to improve the capture of the frames by the scanner’s laser beams.

The computerized models of the plates and screws were then made using the Blender software, by correcting edge flaws, overlaps and gaps in the mesh of the scanned files. The scale was also adjusted by comparing the results obtained in Blender’s dimension measurement tool with the manual measurement of the plates using a digital caliper.

After adjusting the files, they were saved as STL files and imported into the Meshmixer software, where all the necessary modeling was carried out so that the plates matched the geometric shape of the digitally osteotomized jaws, in such a way that they corresponded to the bending limitations of the physical titanium devices.

-Joining and repairing tomographic images, plates and screws

Once the tomographic images, plates and screws had been modeled, these files were merged to correct any persistent flaws in the mesh, using SpaceClaim software (version 2022 R1), an ANSYS CAD program. This stage was carried out in two stages, the first being correction in facet format, using the tools. Still in facet format, fault repairs were carried out on imperceptible edges and vertices, as well as removal with automatic replacement of incompatible edges and vertices, using the tools available in the “Facets” section, these being - facet verification, auto-correct, holes, intersections and correct tips (sharp corners up to 100º and “Sharp Vertices” up to 220º) - then the objects were converted to solid, and the “Repair” and “Prepare” sessions were used, these being - gaps, missing faces, extra edges, inaccurate edges, short edges, faces with defects and sharp edges up to 100º.

**Figure 1:**
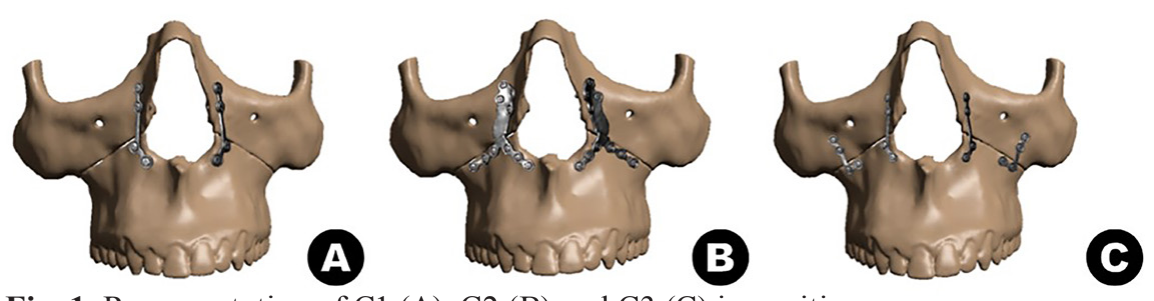
Representation of C1 (A), C2 (B) and C3 (C) in position.

Finally, in Workbench (version 2022 R1), ANSYS’s analysis type structuring program, the previously completed files were imported in “SCDOC” format and the steps were continued (Fig. 1).

-Material Properties and Boundary Conditions

**Figure 2:**
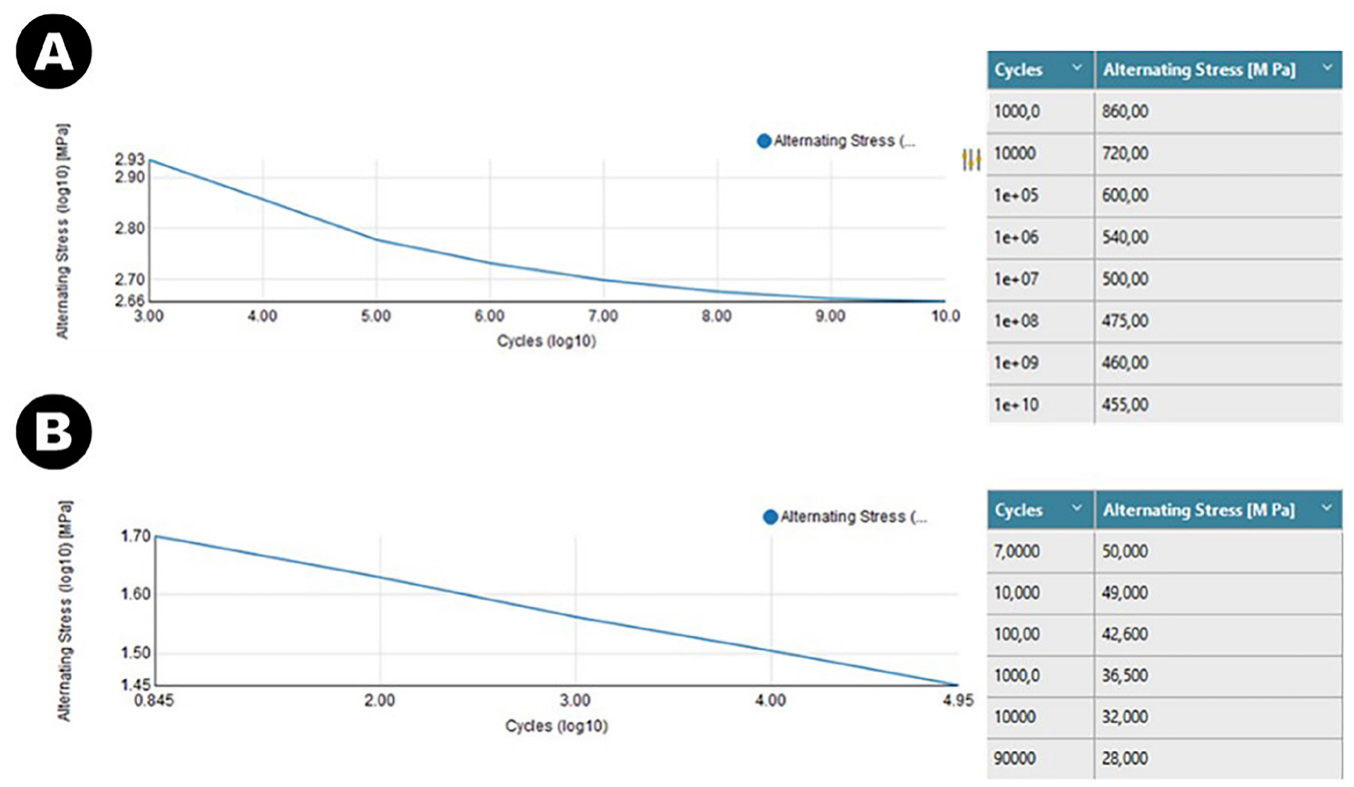
A) data relating to the Ti-6Al-4V charge cycle; B) dados referentes ao ciclo de cargas do osso cortical.

The properties consisted of the Young’s modulus and Poisson’s ratio of the bone material and the miniplate components, for the calculations that were carried out during the simulations. The components were made of a titanium alloy (Ti-6Al-4V). The mechanical properties of cortical bone were used for the bone regions. In addition, the limit stresses in Mega Pascal (MPa) were determined for the Ti-6Al-4V infinite load cycle of 10^6 repetitions (Fig. 2A), as well as for the cortical bone in 9x10^5 repetitions (Fig. 2B).

In this study, it was assumed that the cortical bone in the region of the teeth transfers the masticatory loads to the entire set of miniplate components and the maxilla, according to its internal properties. The connection between the screws and the bone and between the screws and the miniplates was ensured to transmit the load transfer uninterruptedly. It was accepted that the screws were 100% osseointegrated with the surrounding bone tissue. As a result, it was necessary to define in the Mechanical software (version 2022 R1), the program used to define the boundary conditions and carry out the ANSYS analyses, that the plate-screw and screw-bone connections be of the “bonded” type, i.e. there is no movement between the surfaces, a complete surface imbrication. As for the bone-to-bone contact point (posterior region), a “No Separation” contact was defined, i.e. there is only linear displacement in a single plane, which was defined as axial. This type of computational strategy reduces the predictability of the result to the detriment of its execution, i.e. the less computational power the greater the simplification of the boundary conditions.

**Figure 3:**
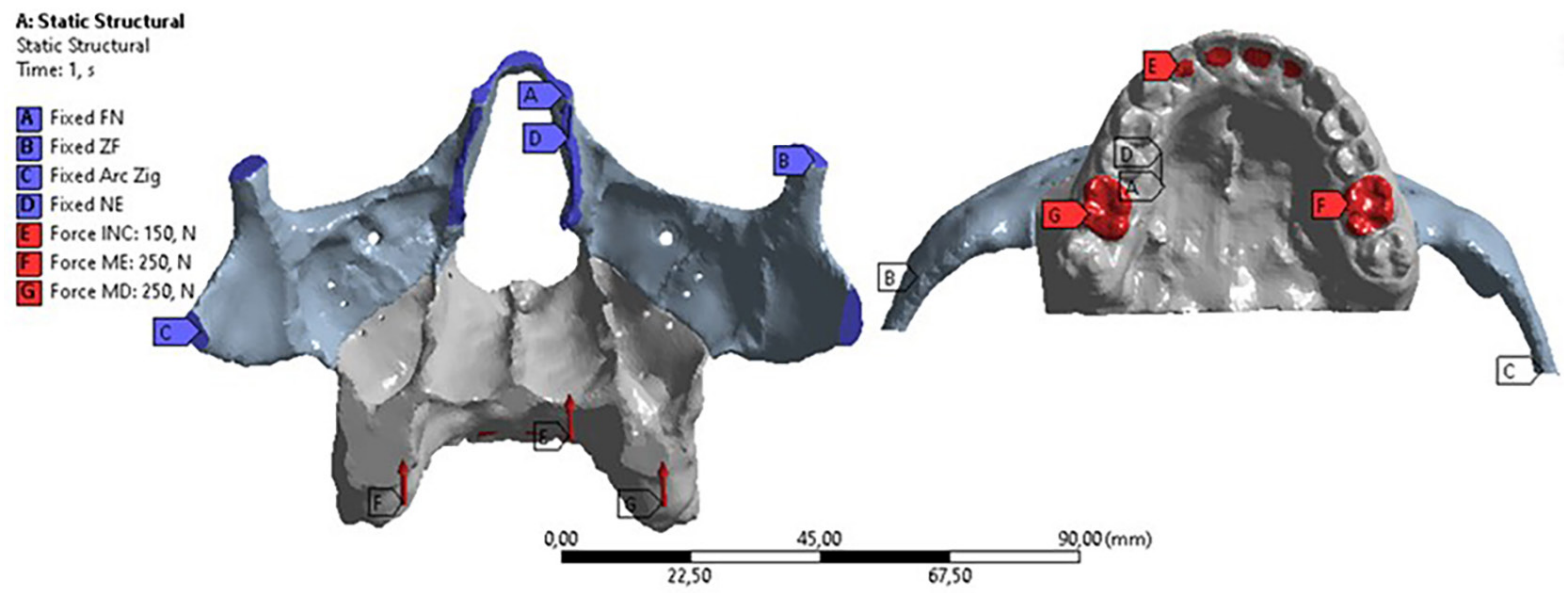
Anchoring areas of the geometric model in space (blue) and areas of application of static vertical forces (red).

The chewing forces were simulated by applying static vertical forces of 250 N to the molar regions (on both sides) and 150 N to the incisor region. In addition, some fixed regions were considered for calculating the deformations of the analyses (Fig. 3).

-Finite Element Meshes

The meshes of the 3 models were made up of tetrahedral finite elements, with their sizes determined by the mesh function based on proximity capture. This function starts from a reference element size (in this study, a reference element size of 3mm was considered) and generates mesh refinements in regions of surfaces or edges that are very close to each other. In addition, local mesh refinements were generated in the component regions, according to the element sizes defined: 0.22mm for screws and 0.16mm for mini-plates.

The mesh was generated to guarantee the quality of the results of the numerical simulations using the finite element method. An Ansys mesh metric was used, called “Element Quality”, which evaluates the quality of the elements based on their deformation in relation to a perfect geometric reference (in this case, a regular tetrahedron), with values between 1, which represents a perfect element, and 0, which represents a well-deformed element. The average metric of the elements in the models was around 0.85.

-Analysis of boundary conditions

To carry out the tests, the following elements were defined: maximum displacement, von mises stress, maximum equivalent stress, minimum equivalent stress, plastic deformation and load cycles.

-Maximum movement

**Figure 4:**
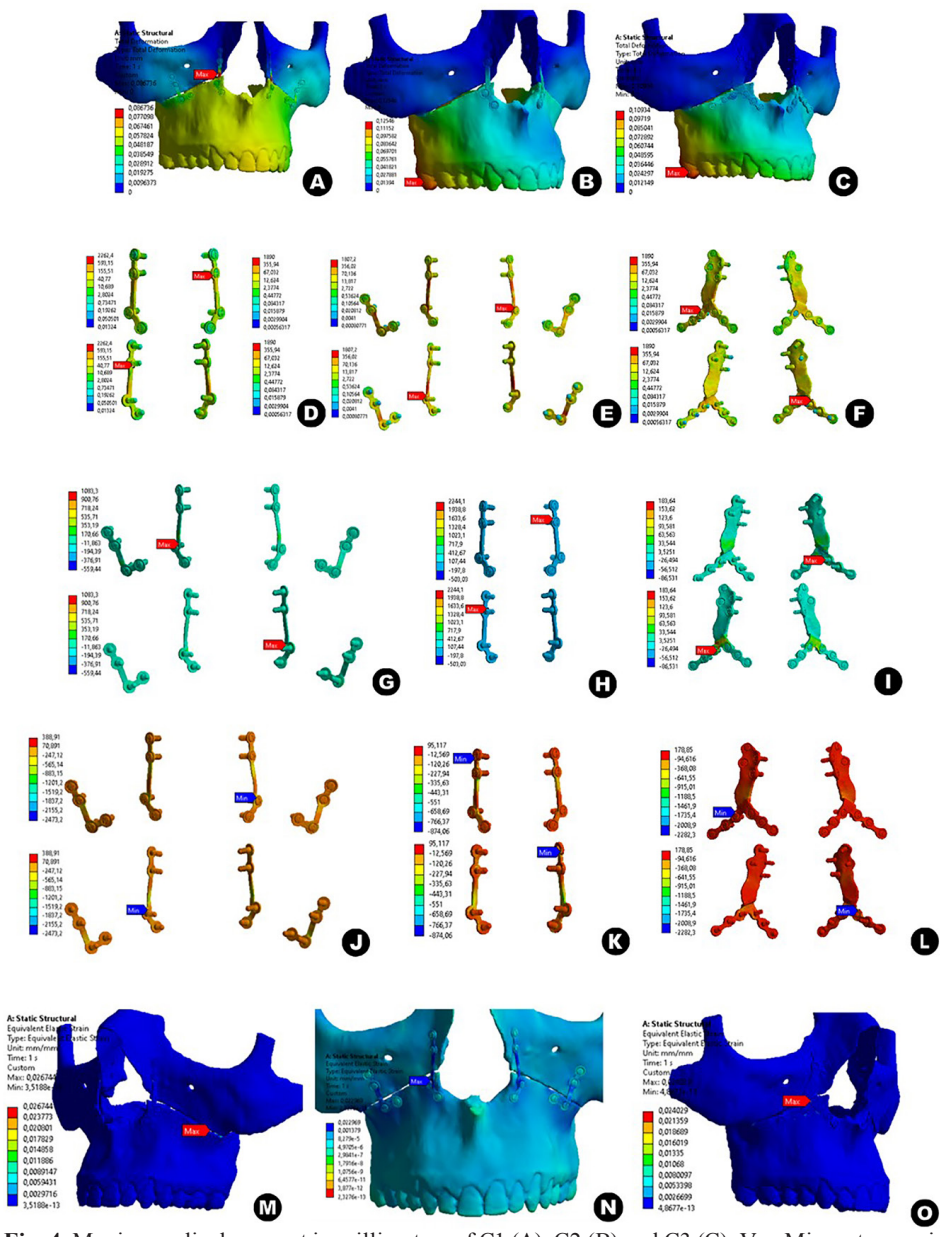
Maximum displacement in millimeters of C1 (A), C2 (B) and C3 (C); Von Mises stresses in MPa of C1 (D), C2 (E) and C3 (F); Maximum equivalent stress in MPa of C1 (G), C2 (H) and C3 (I); Minimum equivalent stress in MPa of C1 (J), C2 (K) and C3 (L); Plastic deformation in millimeters of C1 (M), C2 (N) and C3 (O).

The analysis showed that the maximum displacement in C1 (0.08mm) was in the right anterior region (RA), due to the compressive stresses in the anterior plates, C2 (0.12mm) and C3 (0.1mm) in the right posterior region (RP), due to the overload in the bone-bone contact in the posterior region (Fig. 4A,B,C). It is suggested that this displacement in the anterior region of C1 was the result of the overloads of the anterior plates, described in the following study elements. In relation to the posterior displacement of C2 and C3, it was interpreted as a displacement mostly of the axial plane in the region of bone - bone contact, characterized by a possible lack of posterior anchorage. same pattern of posterior displacement. Following the same pattern described by Li *et al*. (2022) (10), there was a greater concentration of displacement in the posterior molars with a tendency towards anterior projection of the distal segment.

-Von mises stresses

The von Mises stresses at the bend apex (VD) and at the connecting rod (HC) were checked. In C1, VD 762-1058MPa and HC 774-962 MPa. In C2, VD 427-545MPa and HC 299-485MPa. In C3, VD 277-552MPa and HC 162-196MPa (Fig. 4D,E,F). This shows a gradual increase in stresses as the volume of metal decreases, so that even though C1 has 4 attachment points to the anterior plates, its smaller volume means that there is an overload on both posterior faces, but the front is described as having compressive stresses. This behavior is similar to the study described by Huang12, in which the set of 4 “L” plates showed stresses above the yield strength (σ) of Ti-6Al-4V (800Mpa).

As for sets C2 and C3, the stress levels are lower than the σ of Ti-6Al-4V, thus justifying both applications. As described in the studies carried out by Li *et al*. (2022) (10) and Huang *et al*. (2016) (11), the pre-shaped plates showed lower stress and better stress distribution.

-Maximum equivalent stress

The maximum equivalent stress (tension zone) was measured in VD, HC and the bending zone (ZD). In C1, VD 252-285MPa, HC 304-411MPa and ZD 905-958MPa. In C2, VD 105-142MPa, HC 104-158MPa and ZD 96-122MPa. In C3, VD 117-187MPa, HC 61-61MPa and ZD 108-182MPa (Fig. 4G,H,I). As in the comparative study between “L” plates and pre-modeled plates carried out by Li *et al*. (2022) (10) and Huang *et al*. (2016) (11), this element of the analysis elucidates the physical repercussions of the clockwise torsional movement of the plates, so the front faces of the plates showed greater equivalent stresses, since they are being “stretched”. Thus, it can be seen from the data that set C1 showed values above σ in both bending zones of its front plates, thus characterizing the set as a failure. As for assemblies C2 and C3, the stress levels are lower than σ.

-Minimum stress equivalent

The minimum equivalent stress (compression zone) of C1 in VD 883-1068MPa, HD 819-1009MPa and ZD 532-680MPa. In C2, VD 466-645MPa, HC 291-447MPa and ZD 306-507MPa. In C3, VD 285-649MPa, HC 250-201MPa and ZD 271-264MPa (Fig. 4J,K,L). Unlike the previous analysis element, this one provides data on the compression of the material, in the clockwise twisting movement of the plates, the rear faces of the plates showed the minimum equivalent stress, i.e. below zero, negative. The data showed that set C1 had values above σ above the bending zones of its front plates, thus characterizing the failure of the set. As for assemblies C2 and C3, the stress levels were lower than σ.

-Plastic deformation

With regard to plastic deformation, C1 showed changes in RA (0.02-0.022mm/mm) and RP (0.020-0.022 mm/mm), C2 showed changes in RP (0.020-0.022 mm/mm) and C3 showed changes in RA (0.012-0.024mm/mm) and RP (0.022-0.017 mm/mm) (Fig. 4M,N,O). As with maximum displacement, C1 showed stability in the posterior portions, but with high plastic deformation in its anterior plates, i.e. outside its designed dimensional shape. As for C2 and C3, there was plastic deformation at the posterior contact points (bone - bone), but this deformation, due to computational limitations, should be interpreted as inconsistent, since it was necessary to define this region as boned (fixed) contact, where in reality its applicability will be free movement and friction, with the adjacent soft tissues and the fixing action of the osteosynthesis materials limiting its movement.

-Load cycles

In the analysis of load cycles, C1, compression failure in the AR and periosseointegrated surface. In C2 and C3, punctual compression failures in the periosseointegrated surface and in the RP. It is noticeable that most of the areas are in an infinite load cycle, i.e. without failure. However, as previously interpreted, the C1 set showed too much stress in its bending zones and connection rods, either by tension or compression, so it was possible to verify failures in its load cycles in exactly these areas, thus characterizing it as an eminent failure of the osteosynthesis method. As for C2 and C3, they were load efficient. In addition, C3 also showed a better distribution of shears in its threaded contact areas.

## Discussion

Changing the occlusal plane in orthognathic surgery has been studied for some time. According to the studies by Chemello, Wolford and Buschang (1994) (12) and Reyneke *et al*. (2007) (13), regarding the stability of the occlusal plane in bimaxillary surgery, using rigid fixation in mandibular sagittal osteotomies and only in the nasomaxillary pillars of the Le Fort I osteotomy, they report that such movements in both clockwise and counterclockwise directions show good stability in patients without previous pathologies of the temporomandibular joint.

Sursala *et al*. (2020) (14) in their retrospective cohort study, evaluated the stability of occlusal plane changes in clockwise and counterclockwise rotations greater than 2º in patients undergoing bimaxillary surgery with two-point maxillary fixation at the Le Fort I level. The main objectives were to measure cephalometric parameters and the stability of occlusal plane changes at 1 year postoperatively. The authors concluded that rotational movements remained sTable over the 1-year period studied when bilateral nasomaxillary fixation of the Le Fort I osteotomy was performed. This is in agreement with other studies such as Reyneke *et al*. (2007) (13) and Al-Moraissi and Wolford (2016) (15).

In order to evaluate the effectiveness of the different fixation techniques currently available in the literature, finite element analysis can be used to estimate and qualify the mechanical behavior of complex structures under various loading conditions. This method makes it possible to simulate the mechanical stresses and functionality of the various osteosynthesis techniques, providing an understanding of the areas of greatest stress both in the bone tissue and on the surfaces of the fixation material. In this study, three different conFigurations were tested: four-point fixation using L-shaped plates from the 1.5 system (C1), two-point fixation using L-shaped plates from the 2.0 system (C2) and two-point fixation using pre-modeled Lindorf-type plates (C3).

The results showed that C1 had maximum displacement in the anterior region, while C2 and C3 had maximum displacement in the posterior region. This suggests that although C1 offers better posterior anchorage, there is an overload on the anterior plates. Von Mises stresses indicated that C1 exceeded the yield strength of titanium in the anterior plates, suggesting failure of this method. In contrast, C2 and C3 kept the stresses within accepTable limits, demonstrating a more balanced and effective stress distribution.

In the literature, there have already been studies evaluating Linford plates, which, in this study, stood out considerably due to their greater resistance. The study by Lima *et al*. (2022) (16) analyzed Le Fort I osteotomies performed with Linford plates, modified Linford plates and inverted “T” plates. The modified Linford plates and Linford plates provide greater resistance to deformation in Le Fort I osteotomies, while the inverted “T” plates have less rigidity, which reinforces the possibility of using these types of plates, which is in line with the results found.

The biomechanical evaluation of L-shaped plates was also assessed in the study by Esen, Celik, Dolanmaz (2022) (17), where the results showed that the biomechanical behavior of fixation using four mini-plates was better than that of two after applying a 20 N load. In the present study, the result is different, in which the fixation of two L-shaped plates showed better results in terms of the mechanical distribution of the anchorage. One explanation for this may be related to the difference in positioning between the plates and the size of the screws, with 5mm being adopted instead of 2mm, as indicated in the study by Esen, Celik, Dolanmaz (2022) (17).

The osteosynthesis technique using L-shaped plates was also analyzed in the study by Pozzer *et al*. (2017) (2). The study compared stability using pre-modeled T-shaped and L-shaped plates. The L-shaped plates showed greater resistance, especially in relation to vertical loading in the linear displacement range of 1mm/min until peak load and system failure (Pozzer *et al*., 2017) (2). In comparison with the results of the present study, the L-shaped plates showed good results, but when the 2.0mm system was used.

It is worth noting that the finite element analysis method allows different materials and fixation conFigurations to be tested efficiently and economically. Instead of carrying out multiple clinical or laboratory experiments, which are expensive and time-consuming, surgeons can use FEA to carry out several virtual simulations. This makes it possible to optimize fixation techniques even before they are applied to patients, reducing recovery time and increasing the safety of surgical procedures.

The study confirms that fixation techniques with fewer plates (C2 and C3) can be as effective as, or even better than, the traditional four-point fixation method (C1). Fixation with pre-shaped plates (C3) stood out for its better stress distribution and lower mechanical stress. However, more studies are needed to consolidate these findings, especially simulating forces in oblique directions, which are common in the normal masticatory cycle. In addition, the simplifications of the boundary conditions in the computational model may limit the predictability of the results. Finite element analysis remains a powerful tool to guide clinical practice in orthognathic surgery, helping to improve surgical protocols and promote better results for patients.

## Conclusions

Fixation techniques with fewer points, particularly fixation with pre-shaped plates on the nasomaxillary pillars, are highly effective and can be considered a viable alternative to the traditional method of four-point fixation in Le Fort I osteotomy, even in cases of occlusal plane rotation. These techniques not only reduce surgical time and post-operative discomfort, but also maintain the stability and biomechanical efficiency required for a satisfactory surgical result. Further studies are recommended to consolidate these findings and explore the full clinical applicability of these techniques under different loading conditions and vectors.

## Data Availability

The datasets used and/or analyzed during the current study are available from the corresponding author.
